# Comparison of integrin *α*_*v*_*β*_3_ expression with ^68^Ga-NODAGA-RGD PET/CT and glucose metabolism with ^18^F-FDG PET/CT in esophageal or gastroesophageal junction cancers

**DOI:** 10.1186/s41824-023-00162-9

**Published:** 2023-02-01

**Authors:** Matthieu Dietz, Vincent Dunet, Styliani Mantziari, Anastasia Pomoni, Ricardo Dias Correia, Nathalie Testart Dardel, Sarah Boughdad, Marie Nicod Lalonde, Giorgio Treglia, Markus Schafer, Niklaus Schaefer, John O. Prior

**Affiliations:** 1 Nuclear Medicine and Molecular Imaging Department, Lausanne University Hospital, Rue du Bugnon 46, 1011 Lausanne, Switzerland; 2 INSERM U1060, CarMeN Laboratory, University of Lyon, Lyon, France; 3 Department of Diagnostic and Interventional Radiology, Lausanne University Hospital, Rue du Bugnon 46, 1011 Lausanne, Switzerland; 4 University of Lausanne, Lausanne, Switzerland; 5 Department of Visceral Surgery, Lausanne University Hospital, Rue du Bugnon 46, 1011 Lausanne, Switzerland; 6 Clinic of Nuclear Medicine, Imaging Institute of Southern Switzerland, Ente Ospedaliero Cantonale, Bellinzona, Switzerland; 7 Università Della Svizzera Italiana, Lugano, Switzerland

**Keywords:** Esophageal cancer, PET, ^18^F-FDG, ^68^Ga-NODAGA-RGD, Integrin *α*_*v*_*β*_3_, Angiogenesis

## Abstract

**Background:**

The primary aims of this study were to compare in patients with esophageal or esophagogastric junction cancers the potential of ^68^Ga-NODAGA-RGD PET/CT with that of ^18^F-FDG PET/CT regarding tumoral uptake and distribution, as well as histopathologic examination.

**Methods:**

Ten ^68^Ga-NODAGA-RGD and ten ^18^F-FDG PET/CT were performed in nine prospectively included participants (1 woman; aged 58 ± 8.4 y, range 40–69 y). Maximum SUV (SUV_max_) and metabolic tumor volumes (MTV) were calculated. The Mann–Whitney U test and Spearman correlation analysis (*ρ*) were used.

**Results:**

^68^Ga-NODAGA-RGD PET/CT detected positive uptake in 10 primary sites (8 for primary tumors and 2 for local relapse suspicion), 6 lymph nodes and 3 skeletal sites. ^18^F-FDG PET/CT detected positive uptake in the same sites but also in 16 additional lymph nodes and 1 adrenal gland. On a lesion-based analysis, SUV_max_ of ^18^F-FDG was significantly higher than those of ^68^Ga-NODAGA-RGD (4.9 [3.7–11.3] vs. 3.2 [2.6–4.2] g/mL, *p* = 0.014). Only one participant showed a higher SUV_max_ in an osseous metastasis with ^68^Ga-NODAGA-RGD as compared to ^18^F-FDG (6.6 vs. 3.9 g/mL). Correlation analysis showed positive correlation between ^18^F-FDG and ^68^Ga-NODAGA-RGD PET parameters (*ρ* = 0.56, *p* = 0.012 for SUV_max_, *ρ* = 0.78, *p* < 0.001 for lesion-to-background ratios and *ρ* = 0.58, *p* = 0.024 for MTV). We observed that ^18^F-FDG uptake was homogenous inside all the confirmed primary sites (*n* = 9). In contrast, ^68^Ga-NODAGA-RGD PET showed more heterogenous uptake in 6 out of the 9 confirmed primary sites (67%), seen mostly in the periphery of the tumor in 5 out of the 9 confirmed primary sites (56%), and showed slight extensions into perilesional structures in 5 out of the 9 confirmed primary sites (56%).

**Conclusions:**

In conclusion, ^68^Ga-NODAGA-RGD has lower potential in the detection of esophageal or esophagogastric junction malignancies compared to ^18^F-FDG. However, the results suggest that PET imaging of integrin *α*_*v*_*β*_3_ expression may provide complementary information and could aid in tumor diversity and delineation.

*Trial registration:* Trial registration: NCT02666547. Registered January 28, 2016—Retrospectively registered, https://clinicaltrials.gov/ct2/show/NCT02666547.

## Introduction

Angiogenesis is defined as an active process, which regulates the growth of new blood vessels from a pre-existing vascular bed and exerts a prominent role in promoting tumor growth, progression, and metastasis. Integrin *α*_*v*_*β*_3_ is highly expressed on activated endothelial cells of tumor neovasculature and has a key role in tumor angiogenesis (Hood and Cheresh 2002). Arginine-glycine-aspartate (RGD) peptides have a high binding affinity and specificity with integrin *α*_*v*_*β*_3_. As a result, a variety of RGD-based positron emission tomography (PET) imaging agents have been developed to visualize integrin *α*_*v*_*β*_3_ expression (Chen et al. 2016; Dietz et al. 2022). NODAGA-RGDyK, (cyclo[L-arginylglycyl-L-alpha-aspartyl-D-tyrosyl-N6-([4,7-bis(carboxymethyl)octahydro-1H-1,4,7-triazonin-1-yl]acetyl])-L-lysyl]), is a recently developed RGD peptide designed for PET imaging of *α*_*v*_*β*_3_ integrin expression (Jeong et al. 2008). The component NODAGA is a derivate of the NOTA system, which has no influence on receptor-specific binding and possesses high binding properties for radiometals with a ion radius like ^68^Ga (Knetsch et al. 2011). ^68^Ga-NODAGA-RGDyk has favorable biokinetics and safety profile (Buchegger et al. 2011; Gnesin et al. 2017).

Esophageal cancer is the seventh most common cancer worldwide and accounts for more than half a million deaths each year (Bray et al. 2018). The incidence of esophageal squamous cell carcinoma (SCC), the most common histologic type, has been stable, whereas there is an increasing number of esophageal and esophagogastric junction (EGJ) adenocarcinomas in Western countries (Arnold et al. 2020). Angiogenesis was identified as a poor prognosis marker in esophageal cancer (Lurje et al. 2010). Ramucirumab, a vascular endothelial growth factor-receptor 2 (VEGFR-2) antibody, as a single agent or in combination with paclitaxel, is included as an option for second-line or subsequent therapy for patients with metastatic disease (Ajani et al. 2019; Fuchs et al. 2014). However, more data are needed to ascertain whether the addition of such anti-angiogenic therapy to other first-line chemotherapy regimens can improve overall survival (Ajani et al. 2019; Fuchs et al. 2019; Wilke et al. 2014). Currently, there are no validated biomarkers to select patients for anti-angiogenic therapy. Thus, imaging angiogenesis could be crucial to prescreen patients who will benefit from anti-angiogenic therapy.

We hypothesized that the molecular imaging visualization of integrin *α*_*v*_*β*_3_ expression using ^68^Ga-NODAGA-RGD PET/CT could be valuable in exploring esophageal or EGJ malignancies. The primary aims of this study were, first, to compare in patients with esophageal or EGJ cancers the potential of ^68^Ga-NODAGA-RGD PET/ CT with that of ^18^F-FDG PET/CT regarding tumoral uptake and distribution, as well as histopathologic examination, and second, to evaluate quantitative functional imaging parameters from ^68^Ga-NODAGA-RGD PET/CT as potential prognostic markers for disease-free survival (DFS).

## Methods

### Participants

This study was approved by the ethics commission Vaud (protocol CER-VD #120/12) and registered at Clinical-Trials.gov (NCT02666547). Each participant signed a written informed consent form. Inclusion criteria consisted of biopsy-proven esophageal or EGJ cancer, age ≤ 85 years, Karnofsky index ≥ 80%, and signed consent form. Exclusion criteria consisted of pregnancy, lactation period, and age < 18 years.

TNMp or TNMyp (yp denotes the pathological stage after neoadjuvant therapy) stages and DFS (times from the date of scans to the first date of disease recurrence or death) were recorded, according to the criteria of the seventh edition of the Cancer Staging Manual of the American Joint Committee on Cancer. Recurrence was defined as the appearance of one or more new lesions confirmed by imaging or by cytologic or pathological evaluation. Pathology or follow-up examinations were assessed as ground truth in correlation with PET scans.

### PET/CT acquisitions

All the enrolled participants underwent ^68^Ga-NODAGA-RGD and ^18^F-FDG PET/CT using a single dedicated PET/CT scanner (Discovery 690 TOF; GE Healthcare, Waukesha, WI, USA). The same procedure for both ^68^Ga-NODAGA-RGD and ^18^F-FDG PET/CT was used for data acquisition. A pregnancy test was done before the scan in women of childbearing age. Acquisitions were performed with 3 min per bed position. PET data were reconstructed using OSEM (3 iterations, 16 subsets). Vertex to mid-thigh unenhanced CT was acquired for attenuation correction (120 kV, 60 mA, 0.8 s/rotation, pitch 0.9, CTDI 4.54 mGy). The axial resolution was full width at half maximum of 4.7 mm, at 1 cm from the center of the field of view. The mean positron ranges of ^18^F and of ^68^Ga are 0.6 mm and 2.9 mm, respectively.

For ^68^Ga-NODAGA-RGD PET/CT, participants were injected with ^68^Ga-NODAGA-RGDyk. PET/CT images were acquired 59.6 ± 3.5 (range 57–69) min after intravenous administration of 197.5 ± 19.0 (range 165–218) MBq ^68^Ga-NODAGA-RGDyk in an antecubital vein followed by 10 mL of 0.9% NaCl solution.

For ^18^F-FDG PET/CT, participants fasted at least 6 h. Blood glucose levels were checked before ^18^F-FDG administration and were confirmed to be < 8.3 mmol/L. PET/CT images were acquired 62.4 ± 6.1 (range 55–72) min after intravenous injection of 243.5 ± 54.8 (range 155–360) MBq ^18^F-FDG in an antecubital vein followed by 10 mL of 0.9% NaCl solution. The time interval between ^68^Ga-NODAGA-RGD PET/CT and ^18^F-FDG PET/CT was 4.9 ± 2.6 (range 1–9) days.

### Image analysis

PET images were analyzed based on standardized uptake value (SUV) measurements in both data sets (^68^Ga-NODAGA-RGD and ^18^F-FDG), using a workstation equipped with dedicated analysis software (Syngo.via, VB30, Siemens Healthineers, Erlangen, Germany). Scans were evaluated by two experienced nuclear medicine physicians (JOP and MD), blinded to participant's clinical and histologic information. Any difference of opinion was resolved by a consensus. Through visual analysis, positive uptake was identified as areas of focal increase in contrast to the surrounding normal tissue. For the calculation of maximum SUV (SUV_max_) and of metabolic tumor volumes (MTV), circular regions of interest were drawn around tumor lesions with focally increased uptakes in transaxial slices and automatically adapted to 3-D volumes of interest (VOI) delineated around lesions using 60% SUV_max_ thresholds. Lesion-to-background ratios were computed. For the definition of the background, 10-mm-radius circular volumes of interest were drawn in the right atrium (blood pool activity), and the SUV_mean_ was recorded.

The locations of the maximum uptake pixel within primary sites were visually identified in both data sets (^68^Ga-NODAGA-RGD and ^18^F-FDG), and the distance in millimeter (mm) between them was measured.

### Histopathology

Histopathological analysis of tissues obtained from biopsies or resected surgical specimens was based on pathology reports.

### Statistical analysis

The statistical analysis was performed using R version 4.0.3 (R Foundation for Statistical Computing, Vienna, Austria). We assessed the distribution of data with the Shapiro–Wilk test. Continuous parametric variables were expressed as mean ± SD. Nonparametric data were presented as median [interquartile range] and compared using the Mann–Whitney U test. Spearman correlation analysis (*ρ*) was used to evaluate potential interrelation between tracers uptake parameters. Cox’s proportional hazards regression analysis was used to assess the effects of covariates on survival times. A *p* value of < 0.05 was considered statistically significant.

## Results

### Participants

In total, ten ^68^Ga-NODAGA-RGD and ten ^18^F-FDG PET/CT were performed in nine prospectively included participants (1 woman; aged 58 ± 8.4 y, range 40–69 y). Participant’s characteristics are summarized in Table 1. Six had adenocarcinoma, and three had squamous cell carcinoma.

**Table 1:** Baseline characteristics

N	9
Age, y	58 ± 8.4
Men	8 (89%)
Body mass index (kg/m^2^)	29 ± 4.1
*Tumor location at initial diagnosis—N (%)*	
Esophagus	8 (89%)
Gastroesophageal junction	1 (11%)
*Histologic type—N (%)*	
Adenocarcinoma	6 (67%)
Squamous cell carcinoma	3 (33%)
*Pathological lymph node status—N (%)*	
ypN0	1 (11%)
≥ ypN1	3 (33%)
pN0	3 (33%)
≥ pN1	0
Not known	2 (22%)
*Pathological tumor status—N (%)*	
ypT0	1 (11%)
ypT1 or ypT2	0
ypT3 or ypT4	3 (33%)
pT0	0
pT1 or pT2	2 (22%)
pT3 or pT4	1 (11%)
Not known	2 (22%)
*Histologic grade*	
1 or 2	8 (89%)
3 or 4	1 (11%)
Not assessed	0
*Tumor-cell PD-L1 expression—N (%)*	
< 1%	0
≥ 1%	2 (22%)
Indeterminate or could not be evaluated	7 (78%)
*HER2 status*	
Positive	0
Negative	5 (56%)
Not reported	4 (44%)
ECOG performance-status score—N (%) *	
0	6 (67%)
1	3 (33%)

*ECOG performance-status scores range from 0 to 5, with higher scores indicating greater disability

Previous therapies before the PET evaluation, as well as following therapies after the PET evaluation, are described in Table 2. One participant had an anti-angiogenic therapy (ramucirumab) 44 days before the ^68^Ga-NODAGA-RGD PET/CT.

**Table 2:** Therapy

Participants who received concurrent chemoradiotherapy—*N* (%)	Prior scans	Subsequent scans†
*Chemotherapy Neoadjuvant—N (%)*		
Carboplatin/paclitaxel	1 (11%)	3 (33%)
Cisplatin/fluorouracil	0	1 (11%)
Other	0	0
Radiotherapy in concurrent chemoradiotherapy—N (%)	1 (11%)	4 (44%)
*Radiotherapy dosage, Gray—N (%)*		
< 41.4	0	2 (22%)
< 40	0	0
40– < 41.4	0	2 (22%)
41.4–50.4	1 (11%)	2 (22%)
> 50.4	0	0
Not reported	0	0
*Participants with any therapies—N (%)*		
Surgery	2 (22%)	5 (56%)
Interventional radiology	0	1 (11%)
*Systematic therapy—N (%)*		
Immunotherapy	0	2 (22%)
*Targeted therapy—N (%)*		
Anti-angiogenic therapy	1 (11%)	0
Other systemic anticancer therapy/chemotherapy	2 (22%)	1 (11%)

†Before first outcomes

### Comparison of ^68^Ga-NODAGA-RGD PET and ^18^F-FDG PET data

**Fig. 1:**
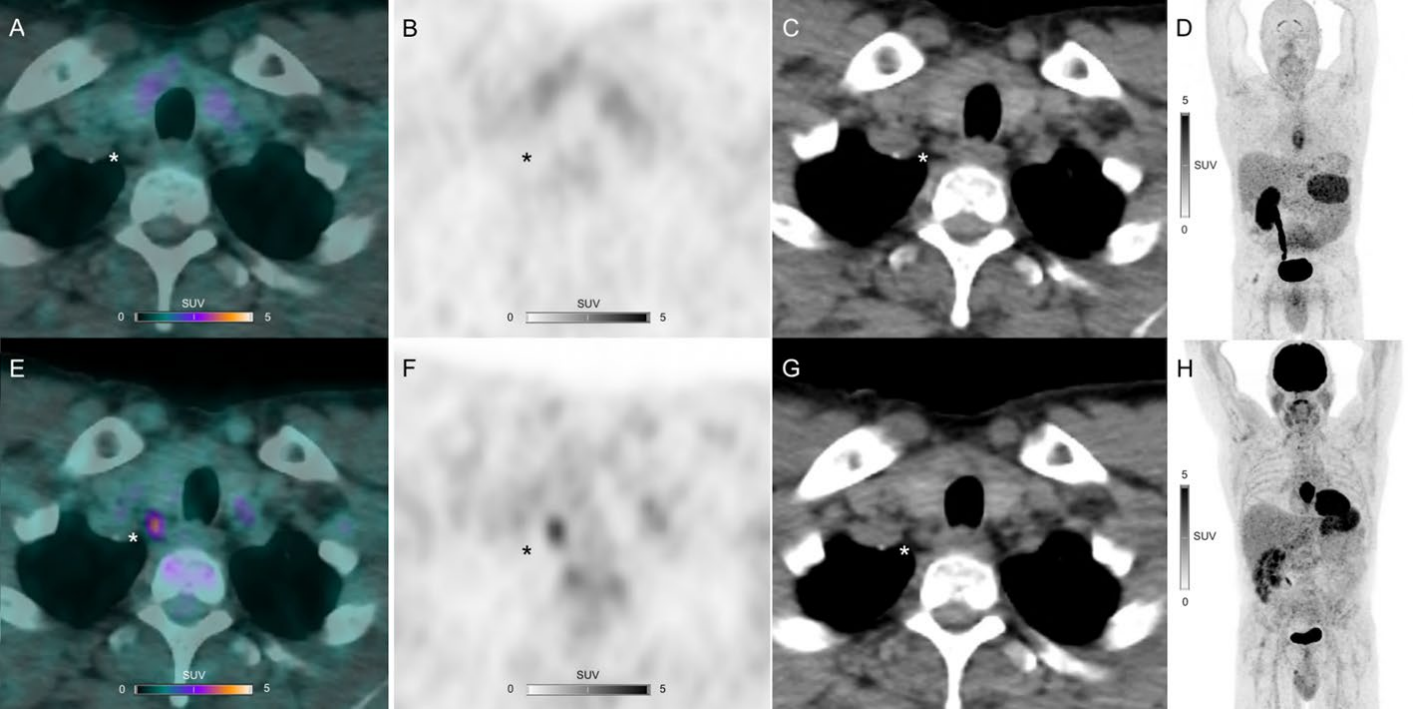
^68^Ga-NODAGA-RGD PET/CT (**A**), PET (**B**, **D**) and CT (**C**) and ^18^F-FDG PET/CT (**E**), PET (**F**, **H**) and CT (**G**) views of an upper paratracheal lymph node metastasis (asterisks) showing no increased ^68^Ga-NODAGA-RGD uptake but intense ^18^F-FDG uptake (SUV_max_ 3.9 g/mL) in a 59-year-old participant with esophageal squamous cell carcinoma

^68^Ga-NODAGA-RGD PET/CT detected positive uptake in 10 primary sites (8 for primary tumors and 2 for local relapse suspicion), 6 lymph nodes and 3 skeletal sites. ^18^F-FDG PET/CT detected positive uptake in the same sites but also in 16 additional lymph nodes and 1 adrenal gland. Data from histology (*n* = 17) or follow-up imaging (*n* = 19) confirmed malignancies, except for a local relapse suspicion (histology proven esophageal candidiasis). An example of an intense ^18^F-FDG uptake in a lymph node metastasis but without increased ^68^Ga-NODAGA-RGD uptake is shown in Fig. 1.

**Fig. 2:**
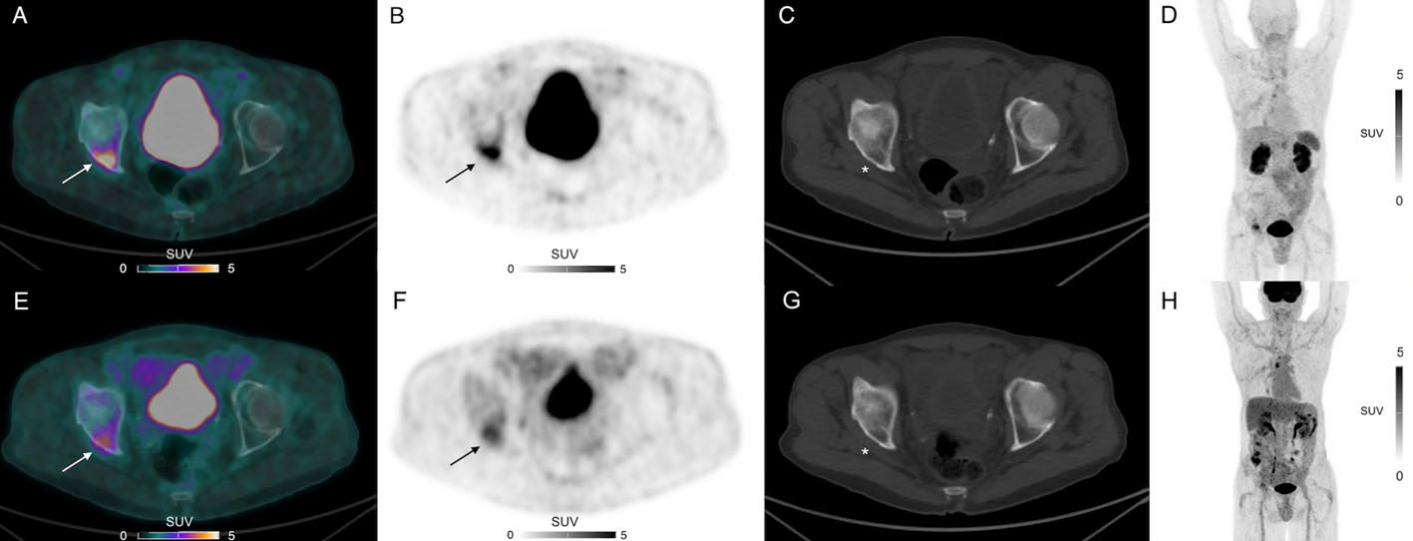
^68^Ga-NODAGA-RGD PET CT (**A**), PET (**B**, **D**) and CT (**C**) and ^18^F-FDG PET CT (**E**), PET (**F**, **H**) and CT (**G**) axial views showing more intense ^68^Ga-NODAGA-RGD uptake compared with ^18^F-FDG uptake (arrows) of an osteolytic lesion (asterisks) of a 63-year-old participant with a metastatic esophageal adenocarcinoma

**Fig. 3:**
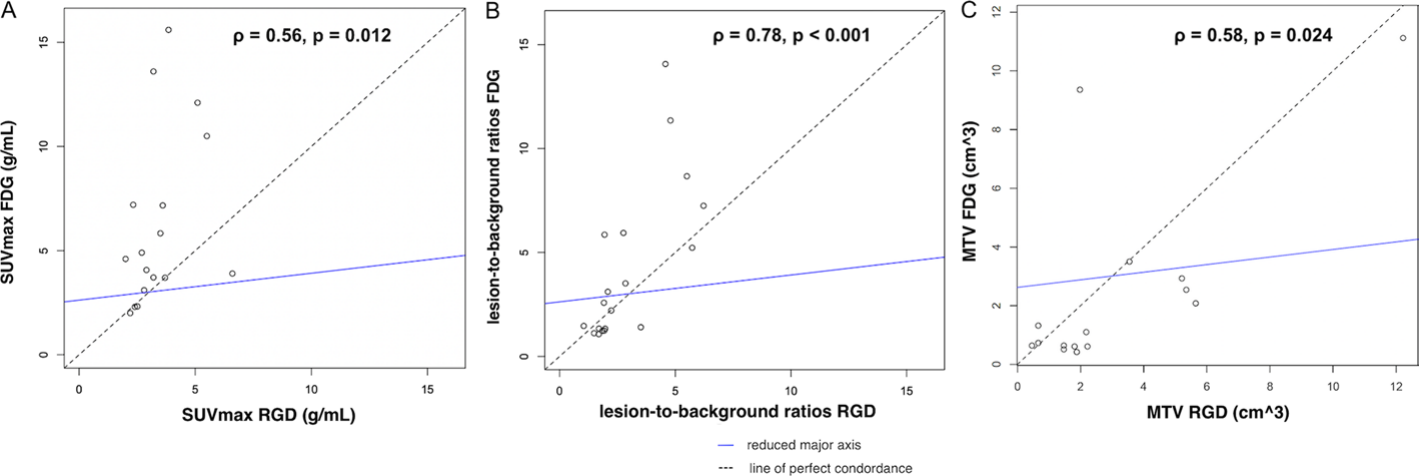
Scatterplots showing positive correlation of SUV_max_ (**A**), lesion-to-background ratios (**B**) and metabolic tumor volumes (MTV) (**C**) of ^18^F-FDG and ^68^Ga-NODAGA-RGD uptake on per-lesion basis

The SUV_max_ measurements of ^68^Ga-NODAGA-RGD and ^18^F-FDG in confirmed lesions are shown in Table 3. On a lesion-based analysis, SUV_max_ of ^18^F-FDG were significantly higher than those of ^68^Ga-NODAGA-RGD (4.9 [3.7–11.3] vs. 3.2 [2.6–4.2] g/mL, *p* = 0.014). Only one participant showed a higher SUV_max_ in an osseous metastasis with ^68^Ga-NODAGA-RGD compared with ^18^F-FDG (SUV_max_ 6.6 vs. 3.9 g/mL, Fig. 2). Blood pool activities of ^18^F-FDG were significantly higher than those of ^68^Ga-NODAGA-RGD (1.8 [1.7–2.2] vs. 1.2 [1.0–1.2] g/mL, *p* = 0.001). When lesion-to-background ratios were compared, no significant difference was found between ^18^F-FDG and ^68^Ga-NODAGA-RGD (2.6 [1.3–5.9] vs. 2.1 [1.9–4.0], *p* = 0.9). Correlation analysis showed moderate to good positive correlation between ^18^F-FDG and ^68^Ga-NODAGA-RGD PET parameters (*ρ* = 0.56, *p* = 0.012 for SUV_max_, *ρ* = 0.78, *p* < 0.001 for lesion-to-background ratios and *ρ* = 0.58, *p* = 0.024 for MTV; Fig. 3).

**Table 3:** Measurements of ^68^Ga-NODAGA-RGD or ^18^F-FDG uptake in confirmed positive uptake

	^68^ Ga-NODAGA-RGD				^18^F-FDG			
All	Median SUVmax (g/mL)	Median Tumor-to blood pool background ratio	Median MTV 60% (cm^3^)	Confirmed Lesions (N)	Median SUVmax (g/mL)	Median Tumor-to blood pool background ratio	Median MTV 60% (cm^3^)	Confirmed Lesions (N)
Primary sites	3.85	4.58	5.21	9	12.1	5.94	2.31	9
*Involved lymph nodes*								
Neck and supraclavicular	NA	NA	NA	NA	4.3	2.39	1.03	1
Mediastinum	3.2	1.93	1.07	5	3.75	1.99	0.73	14
Abdomen-Pelvis	2.33	1.94	1.88	1	5	2.78	0.95	7
All	2.85	1.94	1.47	6	3.96	2.17	0.94	22
*Bone and visceral metastases*								
Bone	3.5	2.85	2.77	3	3.9	1.4	3.5	3
Adrenal gland	NA	NA	NA	NA	5.61	3.38	12.18	1
All	3.5	2.85	2.77	3	4.76	2.39	6.43	4

*NA* not applicable

We incidentally detected a focal increased uptake of ^68^Ga-NODAGA-RGD in the thyroid, which was absent on the ^18^F-FDG PET scan. No further investigation could have been done since the participant died 48 days after the ^68^Ga-NODAGA-RGD PET/CT.

### Uptake patterns within primary lesions

**Fig. 4:**
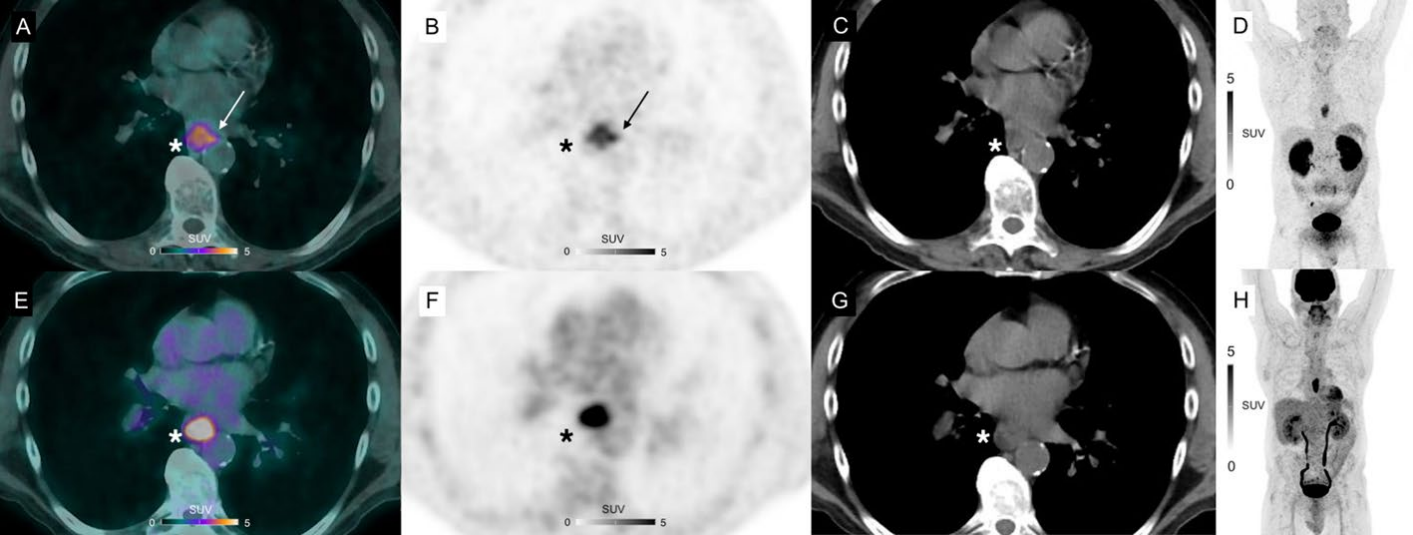
^68^Ga-NODAGA-RGD PET/CT (**A**), PET (**B**, **D**) and CT (**C**) and ^18^F-FDG PET/CT (**E**), PET (**F**, **H**) and CT (**G**) axial views of a primary lesion (asterisks) in a 53-year-old participant with esophageal squamous cell carcinoma. Note intense homogenous uptake in the primary lesion on ^18^F-FDG PET/CT (SUV_max_ 12.1 g/mL, metabolic tumor volume 2.54 cm^3^), whereas the primary lesion demonstrates a different uptake pattern in the corresponding ^68^Ga-NODAGA-RGD PET/CT image: a weaker and more heterogeneous uptake (SUV_max_ 5.1 g/mL), seen mostly in the periphery of the tumor, with a slight extension in perilesional structures (arrows), and a larger metabolic tumor volume (metabolic tumor volume 5.35 cm^3^)

The distribution of both tracers within primary sites was different. We observed that ^18^F-FDG uptake was homogenous inside all the confirmed primary sites (*n* = 9). In contrast, ^68^Ga-NODAGA-RGD PET showed more heterogenous uptake in 6 out of the 9 confirmed primary sites (67%), seen mostly in the periphery of the tumor in 5 out of the 9 confirmed primary sites (56%), and showed slight extensions into perilesional structures in 5 out of the 9 confirmed primary sites (56%). An example of these different uptake patterns is shown in Fig. 4.

The median distance between the maximum uptake locations of both scans was 6.5 [4.5–14] mm. This median distance was greater than the PET/CT scanner resolution and the mean positron ranges of ^18^F and ^68^Ga.

### Effect of pathological tumor status and histologic grade

Both tracers’ SUV_max_ in primary lesions did not correlate with pathological tumor status (dichotomized by status Tp or Typ ≤ 2 vs. Tp or Typ > 2; ^68^Ga-NODAGA-RGD, 3.8 [3.2–5.1] vs. 4.6 [3.7–5.0] g/mL; ^18^F-FDG, 12.1 [10.5–13.6] vs. 16.8 [10.0–17.0] g/mL, *p* ≥ 0.57 for both).

Both tracers’ SUV_max_ in primary lesions did not correlate with pathological tumor status (dichotomized by histologic grade 1 or 2 vs. histologic grade 3 or 4; ^68^Ga-NODAGA-RGD, 3.9 [3.0–5.3] vs. 4.6 g/mL; ^18^F-FDG, 12.1 [7.7–14.6] vs. 16.8 g/mL, *p* ≥ 0.5 for both).

### Outcomes

Over the 825 ± 623 days [range 48–1786] of follow-up, four participants have experienced disease recurrence and two participants died. The median progression-free survival was 308 days. No ^68^Ga-NODAGA-RGD uptake measurement in primary lesions turned out to be a prognostic factor for DFS on univariate analysis (SUV_max_, HR 95% CI 0.44–2.71, *p* = 0.8; Lesion-to-background ratio, HR 95% CI 0.62–2.22, *p* = 0.6; MTV 60%, HR 95% CI 0.83–1.59, *p* = 0.4). Interestingly, the only participant who showed a lesion with a higher SUV_max_ with ^68^Ga-NODAGA-RGD compared with ^18^F-FDG (Fig. 2) experienced disease recurrence 231 days after the ^68^Ga-NODAGA-RGD PET/CT study and died 962 days after, during disease progression with fluorouracil, l-leucovorin, and irinotecan chemotherapy. Nevertheless, statistically, the presence of a lesion with higher SUV_max_ with ^68^Ga-NODAGA-RGD compared with ^18^F-FDG did not turn out to be a prognostic factor for DFS on univariate analysis in the current small cohort (HR 95% CI 0.22–20.5, *p* = 0.5).

## Discussion

We report several notable findings from this prospective preliminary study of ^68^Ga-NODAGA-RGD PET imaging in esophageal or esophagogastric junction cancers. First, the molecular imaging visualization of integrin α_v_β_3_ expression using ^68^Ga-NODAGA-RGD PET/CT has lower potential in the detection of esophageal or esophagogastric junction malignancies compared to the visualization of glucose metabolism with ^18^F-FDG PET/CT. However, ^68^Ga-NODAGA-RGD PET/CT showed different uptake patterns in most primary lesions than ^18^F-FDG PET/CT, and ^68^Ga-NODAGA-RGD uptake was not systematically lower, suggesting that ^68^Ga-NODAGA-RGD may provide complementary information.

The study of molecular imaging of integrin expression focused on esophageal or EGJ malignancies has not previously been well established in the literature. To the best of our knowledge, the only previous study evaluating RGD imaging on the evaluation of esophageal cancer is a prospective study by Zheng et al. investigating the efficacy of [^99m^Tc]3PRGD_2_ on standard gamma cameras (Zheng et al. 2019).

Our finding of a lower detection rate of ^68^Ga-NODAGA-RGD than ^18^F-FDG imaging in detecting malignancies is not unexpected and is consistent with previous other cancer studies in humans. Zheng et al. found a lower sensitivity than ^18^F-FDG imaging for detecting small esophageal metastatic lesions in lymph nodes. Beer et al. found a lower sensitivity for lesion detection for ^18^F-galacto-RGD PET as compared to ^18^F-FDG PET in eighteen cancer patients, mostly with non-small cell lung cancer (Beer et al. 2008). Haubner et al. demonstrated no increased uptake of ^68^Ga-NODAGA-RGD in hepatocellular carcinoma compared with the background liver tissue (Haubner et al. 2016). In contrast, ^18^F-FPPRGD_2_ showed higher sensitivity and specificity than ^18^F-FDG in a preliminary PET study on breast cancer by Iagaru et al. (Iagaru et al. 2014).

The finding of a significantly higher uptake with ^18^F-FDG than with ^68^Ga-NODAGA-RGD in positive lesions is also not surprising, and consistent with previous studies in humans (Beer et al. 2008; Durante et al. 2020). To explain this difference in tracer uptake, Beer et al. argued that ^18^F-galacto-RGD binds predominantly to endothelial cells, with a substantially smaller number than the number of FDG-avid tumor cells (Beer et al. 2008). As both ^18^F-Galacto-RGD and ^68^Ga-NODAGA-RGD demonstrated similar preclinical results (Pohle et al. 2012), this same theory could be applied to our study. However, a significantly lower tracer uptake does not necessarily mean a lower lesion-to-background ratio. In the present study when lesion-to-background ratios in positive lesions were compared, no significant difference was found between ^18^F-FDG and ^68^Ga-NODAGA-RGD. Same results were shown in a prospective study by Minamimoto et al. (2015). By comparing ^18^F-FPPRGD_2_ and ^18^F-FDG uptake values in various non-esophageal cancer patients, those authors showed no significant difference in tumor-to-background ratios between both tracers. The low RGD-based tracer uptake in several areas such as the lung, muscles, fat, the brain, or the myocardium could be an advantage for both qualitative and quantitative evaluation of thoracic, breast or brain lesions (Beer et al. 2008; Minamimoto et al. 2015), or for non-oncological applications such as cardiovascular imaging or inflammatory diseases (Dietz et al. 2021, 2022; Ebenhan et al. 2021; Zhu et al. 2014).

An encouraging finding is the fact that an osteolytic malignant lesion showed a clearly more intense ^68^Ga-NODAGA-RGD uptake as compared to ^18^F-FDG. This result is consistent with preclinical data, which supported that RGD-based PET tracer has the potential to effectively image bone metastases, especially in osteolytic metastases, by targeting of the *α*_*v*_*β*_3_ integrin on osteoclasts and the proinflammatory cells involved at the bone metastatic site (Wadas et al. 2009). In a pilot prospective study of ^18^F-Alfatide II for detection of skeletal metastases in humans, Mi et al. showed high positive predictive value in the detection of bone metastases, with high lesion-to-background contrast (Mi et al. 2015). This observation is in alignment with the hypothesis that RGD-based imaging may provide complementary information in imaging cancer patients.

We strongly believe that the complementary information provided by molecular imaging of *α*_*v*_*β*_3_ expression could be clinically relevant. Integrins, especially the *α*_*v*_*β*_3,_ are associated with tumor angiogenesis and the blockade of integrin signaling has been shown to inhibit tumor growth, angiogenesis, and early metastasis (Liu et al. 2008). Despite the intriguing concept of anti-angiogenesis, initially described by Folkman et al. (1971), the real therapeutic breakthrough of this treatment never really held its promise and induced only very modest improvements in overall survival (Ribatti et al. 2019). One of the most prominent trials addressing *α*_*v*_*β*_3_/*α*_*v*_*β*_5_ inhibition was the CENTRIC trial [Celengitide, Merck KGaA, Darmstadt, Germany] in glioblastoma delivering negative results (Stupp et al. 2014).

The escape mechanisms of cancer against anti-angiogenic treatments are manifold but one key element of resistance is the heterogeneity of neoplastic endothelial cells (Montemagno and Pagès 2020). ^68^Ga-NODAGA-RGD PET/CT is a noninvasive, holistic imaging of tumor angiogenesis and could play a pivotal role in identifying patients which have greatest benefit from anti-angiogenetic therapy. This hypothesis is supported by data from the CORE study, where glioblastoma patients with higher *α*_*v*_*β*_3_/*α*_*v*_*β*_5_ had significantly better outcomes (Nabors et al. 2015). This clearly demonstrates the need of biomarkers to select patients and find an optimal treatment window for patients receiving anti-angiogenic treatments. Especially functional imaging depicting angiogenic targets as *α*_*v*_*β*_3_ could greatly help to select patients and an optimal time window for such treatments. ^68^Ga-NODAGA-RGD might even serve as theranostic imaging marker followed by therapeutic beta-particle based radioligand therapy (Bozon-Petitprin et al. 2015). Such radioligand therapy could potentially overcome the shortcoming of classical anti-angiogenic therapy by a crossfire effect anticipating the heterogeneity in endothelial cells.

Furthermore, *α*_*v*_*β*_3_ integrin is involved in the epithelial–mesenchymal transition, which plays a pivotal role in the very early stages of tumorigenesis and tumor implantation (Kariya et al. 2021; Liu et al. 2017). ^18^F-FDG PET is widely accepted as preferred method for initial tumor staging in esophageal cancer. ^68^Ga-NODAGA-RGD with its extensions of uptake into perilesional structures could help to delineate the pre-tumoral and pre-metastatic niche. In the near future, local procedures like surgical resection of radiotherapy in esophageal cancer might use ^68^Ga-NODAGA-RGD uptake to optimally plan their resection margins or radiotherapy fields. Further investigations would be still required in the future to elucidate the potential role of ^68^Ga-NODAGA-RGD in esophageal cancer management.

### Limitations

There exist some limitations in our study. ^68^Ga-NODAGA-RGD uptake was not prognostic for any of the investigated endpoints, but our number of participants is not large enough. The limited statistical power may also explain the absence of significant results in subgroup analysis for different pathological tumor status or histologic grade. Immunohistochemistry tests were not performed to assess the correlation between integrin *α*_*v*_*β*_3_ expression and ^68^Ga-NODAGA-RGD uptake, which has been demonstrated in several animal and clinical studies (Chen et al. 2016; Jeong et al. 2008).

## Conclusion

In conclusion, ^68^Ga-NODAGA-RGD has lower potential in the detection of esophageal or esophagogastric junction malignancies compared to ^18^F-FDG. However, the results suggest that ^68^Ga-NODAGA-RGD may provide complementary information, indicating that PET imaging of integrin α_v_β_3_ expression could aid in tumor diversity and delineation.

## Data Availability

The datasets used and/or analyzed during the current study are available from the corresponding author on reasonable request.
